# Validation of a novel western blot assay to monitor patterns and levels of alpha dystroglycan in skeletal muscle of patients with limb girdle muscular dystrophies

**DOI:** 10.1007/s10974-024-09670-y

**Published:** 2024-04-18

**Authors:** Thulashitha Rajasingham, Hector M. Rodriguez, Andreas Betz, Douglas M. Sproule, Uma Sinha

**Affiliations:** 1 Department of Preclinical/Clinical Pharmacology, ML Bio Solutions, a BridgeBio company, Palo Alto, USA; 2 Department of Clinical Development, ML Bio Solutions, a BridgeBio company, Palo Alto, USA

**Keywords:** Alpha dystroglycan, Biomarker, Fluorescence, Glycosylation, Limb girdle muscular dystrophy, Western blot

## Abstract

The cell membrane protein, dystroglycan, plays a crucial role in connecting the cytoskeleton of a variety of mammalian cells to the extracellular matrix. The α-subunit of dystroglycan (αDG) is characterized by a high level of glycosylation, including a unique O-mannosyl matriglycan. This specific glycosylation is essential for binding of αDG to extracellular matrix ligands effectively. A subset of muscular dystrophies, called dystroglycanopathies, are associated with aberrant, dysfunctional glycosylation of αDG. This defect prevents myocytes from attaching to the basal membrane, leading to contraction-induced injury. Here, we describe a novel Western blot (WB) assay for determining levels of αDG glycosylation in skeletal muscle tissue. The assay described involves extracting proteins from fine needle tibialis anterior (TA) biopsies and separation using SDS-PAGE followed by WB. Glycosylated and core αDG are then detected in a multiplexed format using fluorescent antibodies. A practical application of this assay is demonstrated with samples from normal donors and patients diagnosed with LGMD2I/R9. Quantitative analysis of the WB, which employed the use of a normal TA derived calibration curve, revealed significantly reduced levels of αDG in patient biopsies relative to unaffected TA. Importantly, the assay was able to distinguish between the L276I homozygous patients and a more severe form of clinical disease observed with other *FKRP* variants. Data demonstrating the accuracy and reliability of the assay are also presented, which further supports the potential utility of this novel assay to monitor changes in ⍺DG of TA muscle biopsies in the evaluation of potential therapeutics.

**Supplementary Information:**

The online version contains supplementary material available at 10.1007/s10974-024-09670-y.

## Introduction

Alpha-dystroglycan (αDG) is a highly glycosylated peripheral membrane protein which is an integral part of the dystrophin-glycoprotein complex (Nickolls and Bonnemann 2018; Barresi and Campbell 2006; Le et al. 2018; Han et al. 2009). It is encoded by the *DAG1* gene and is ubiquitously expressed. The *DAG1* gene translates into a single polypeptide which is then cleaved into two subunits: αDG and βDG. αDG is secreted into the extracellular space but interacts non-covalently with βDG, which remains anchored in the membrane (Kanagawa et al. 2004). αDG is found in cells of various tissues including skeletal muscle, nervous system, digestive tract, kidney, skin, and reproductive organs where it provides a crucial link between the cytoskeleton, through its indirect binding to dystrophin, and the basal lamina. In skeletal muscles, αDG interacts with extracellular ligands, including laminin-α2, a significant functional protein of the extracellular matrix crucial for muscle function and structure (Balci-Hayta et al. 2018). This interaction confers structural stability to the sarcolemma during contraction. αDG has a mucin domain rich in O-linked glycans, such that carbohydrates constitute over half of the glycoprotein’s mass (Kanagawa et al. 2004). In this heavily glycosylated mucin site, 3 specific threonine sites, T317, T319, and T379, are modified for matriglycan synthesis (Sheikh et al. 2022; Inamori et al. 2012). Reduced levels of matriglycan, result from mutations in at least seventeen genes causing a variety of complex diseases affecting both muscular and nervous systems (Yoshida-Moriguchi and Campbell 2015; Hewitt 2009; Barresi and Campbell 2006; Martin 2005).

A novel western blot assay was developed to monitor changes in αDG in the skeletal muscle. The assay is based on multiplexed detection using fluorescence. For detection, the monoclonal antibody IIH6C4 that recognizes a specific functional epitope of the matriglycan chain of glycosylated αDG (a tandem repeat of xylose and glucuronic acid was used; the matriglycan is the specific recognition site for laminin α2 (Ervasti and Campbell 1993). Therefore, IIH6C4 binding uniquely acts as a proxy for molecular functional assessment of αDG. Specifically, the presence of IIH6C4-reactive matriglycan on αDG indicates that the glycosylation pathway is intact, and that the protein can interact with the extracellular matrix, thereby establishing the clinical relevance of this antibody (Stevens et al. 2013). In addition, the core protein is detected using the antibody AF6868, regardless of αDG's glycosylation status. This approach of simultaneous detection of both core and glycosylated protein from the same sample interrogates both the amount and status of glycosylation of αDG. The assay incorporates a calibration curve using normal donor TA for determination of relative amounts of αDG. Additionally, quality controls are used to monitor the assay’s performance. The precision, linearity, specificity, sensitivity, and consistency in detection of alterations in αDG levels are reported. In addition to the quantitative aspect, this assay has the potential to provide information on molecular changes the protein undergoes over time or upon therapeutic intervention in patients with α-dystroglycanopathies.

The applicability of the assay was demonstrated using skeletal muscle biopsies from patients with LGMD2I/R9, a disease caused by mutations in fukutin-related protein gene (*FKRP*). FKRP is a glycosyltransferase that adds a critical ribitol 5-phosphate to the growing matriglycan chain in αDG during its functional maturation. FKRP’s role in αDG glycosylation is significant in brain and striated muscles' function and mutations can disrupt this process, leading to various forms of muscular dystrophy. Overall, this bioassay and the use of glycosylated αDG as a potential biomarker serve as a powerful tool to monitor the efficiency of therapeutic intervention and longitudinal responses to disease-modifying therapies of α-dystroglycanopathies.

## Materials and methods

### Antibodies and proteins

To detect both glycosylated and core αDG, anti-αDG clone IIH6C4 (Sigma, St. Louis, MO) and anti-human DG AF6868 (R&D System, Minneapolis, MN) primary antibodies were used, respectively. As secondary antibodies IRDye 680 linked Goat anti-Mouse IgM (LI-COR) and Alexa Fluor 790 linked Mouse anti-Sheep IgG (Jackson Immuno Research, PA) were used for detecting IIH6C4 and AF6868 respectively. Chameloeon Duo (LI-COR) was used as the molecular weight marker.

### Tibialis anterior (TA) biopsy collection

Biopsies from patients with an LGMD2I/R9 diagnosis were collected using fine needle aspiration (FNA) (14-gauge × 6 cm; SuperCore™ Instrument; Cincinnati, OH, USA), a minimally invasive biopsy technique,  in an Institutional Review Board (IRB) approved Natural History Study (MLB-01–001; NCT04202627). This study was conducted in accordance with the principles laid out in the Declaration of Helsinki. All procedures involving human subjects were performed with respect for their rights and dignity. Written and oral informed consent was obtained from participants, parents, or legal guardians before enrollment into this study, and their anonymity and confidentiality were preserved throughout the study. Up to three serial biopsy cores were obtained per visit and pooled for analysis. For controls, biopsies from six normal individuals, without muscular dystrophy, cardiac disease, or diabetes, were obtained from BioIVT (Westbury, NY, USA). Tibialis Anterior from a transgenic mouse with an FKRP-P448L mutation was a gift from Dr. Qi Long Lu, Atrium Health.

### Sample homogenization and considerations for high molecular weight protein extraction

For homogenization, multiple cores of frozen TA biopsies were combined in a 2-mL tube (TissueLyser-safe). A lysis buffer containing 125 mM Tris, 10% glycerol, 10% β-mercaptoethanol, urea, SDS and 1 mM of the protease inhibitor AEBSF was then added to the tissue, along with a single stainless-steel bead. The sample tubes were then processed using a TissueLyser II (Qiagen, Venlo, Netherlands). For storage, to maintain protein stability over time, excess lysate was divided into multiple aliquots and flash frozen at −80 °C to minimize freeze/thaw cycles. Ongoing studies have shown that the protein is stable for at least 18 months.

### Dot blot assay

The specificity and cross-reactivity of both primary and secondary antibodies were tested using a dot blot assay. Briefly, TA lysate was prepared at three different concentrations: 30, 15, and 5 µg of total protein in 1xPBS. The diluted lysate was drop-pipetted onto six separate nitrocellulose membranes following the same layout and dried at room temperature followed by a rinse with 1xPBS for membrane re-activation. To detect nonspecific binding and cross-reactivity, the membranes were blocked for 1 h at room temperature and then sequentially incubated with primary antibodies (overnight at 4 °C) and secondary antibodies (for 1 h at room temperature). Combinations used for testing cross-reactivity and nonspecific binding are listed in Fig. 2b.

### Preparation of calibration controls, quality controls and patient samples

Calibration standards and positive quality controls were freshly prepared using normal (non-affected) donor TA lysate (BioIVT). Control TA standards for a seven-point calibration curve were prepared in incremental amounts of total protein. The high positive control (HPC) was prepared using normal TA lysates mixed with lysis buffer and sample loading buffer to a load of 10 µg per gel. This mixture was heat denatured, divided into single-use aliquots, and frozen between −90˚C and −65˚C for use in validation and sample analyses. The low positive control (LPC) was prepared similarly at a load of 5 ug. The negative controls (NC) were prepared from a commercially available recombinant human DG (rhDG; R & D systems, Minneapolis, MN) expressed in NS0 cells at a concentration of 10 ng in PBS. LGMD2I/R9 lysate samples were diluted in matrix buffer (the same lysis buffer) to achieve a total protein concentration of 50 µg for L276I homozygous samples and 70 µg for other *FKRP* genotype samples. These samples were prepared for loading by spiking with 0.001% Bromophenol blue in a final volume of 15 µL.

### SDS-PAGE and western blot analysis

All samples were boiled and loaded onto a 4–20% Tris–Glycine polyacrylamide gel (Novex, Invitrogen). Proteins were separated and then transferred onto a nitrocellulose membrane (Invitrogen) using the gel transfer device, iblot2 (Invitrogen, Carlsbad, CA). Nonspecific binding sites on the membrane were blocked with Intercept blocking buffer (LI-COR, Lincoln, Nebraska) prior to incubation with a mixture of target-specific primary antibodies. These included the anti-α-DG antibody clone IIH6C4 at 1:1000 dilution, which is specific for glycosylated αDG, and human αDG antibody AF6868 at 0.12 µg/mL, specific to αDG regardless of its glycosylated status. The primary antibodies were incubated overnight on a rocking platform at 4 ˚C. Following a series of washes with 1X TBST (tris buffered saline with tween) to remove unbound primary antibodies, the membranes were incubated with a mixture of secondary antibodies. Each blot was incubated with the secondary antibodies at a 1:5000 dilution in 25 mL antibody diluent at room temperature. Membranes were then incubated with a mixture of appropriate near-infrared dye 680RD (700 nm emission) and Alexa Fluor 790 (800 nm emission) secondary antibodies. The membranes were washed again to remove unbound secondary antibodies and images were captured using an Odyssey imager (LI-COR, Lincoln, Nebraska).

### Region of interest (ROI)

Densitometric measurements are taken within an ROI to quantify the amount of protein defined by the molecular weight marker (Butler et al. 2019). For lanes with calibration curve samples, an ROI corresponding to 125 to 260 kDa is used to capture signals of fully glycosylated αDG protein of 157 kDa. For non-calibration curve samples, which includes patient samples and HPC, a broader ROI is used from 70 to 260 kDa. This ROI was chosen as patient samples are expected to have varying degrees of αDG glycosylation, resulting from the defective FKRP, and thus are expected to migrate in this molecular weight range. Lower MW, less glycosylated forms of αDG, are gated using the DG antibody AF6868.

Table 1 summarizes the ROIs for glycosylated αDG and core αDG signals which are detected simultaneously on separate fluorescence channels (700 and 800 nm, respectively) and analyzed using Image Studio™ software by examining boxed regions of the membranes with the guidance of the molecular weight ladder (Chameleon Duo, Licor).

**Table 1:** Regions of interest used for data collection

	Molecular weight reference Range, kDa	Lowest anchor point, kDa
Calibration curve samples	125–260	125
QC control (HPC)	70–260	70
Patient samples	70–260	70

### Background correction

For each ROI box drawn, sample signal is corrected for background using Image Studio software according to the following equation:

$$\mathrm{Corrected\, Signal}=\mathrm{Total \,Signal}-({\text{Background}}\times \mathrm{Area\, of\, ROI}),$$

where background is the sum of pixel intensities of the border selected.

### Determination of αDG levels

For each blot, two calibration curves are generated for the 700 and 800 nm channels, which correspond to glycosylated and core αDG, respectively. These curves are created using a 7-point dilution series ranging from 1.25 to 25 µg of lysate from the selected normal donor, TA1. Raw fluorescence signals from the 700 and 800 nm channels for each patient’s biopsy lysate are interpolated to their corresponding calibration curve. This allows for determination of relative TA equivalent levels of glycosylated and core αDG, respectively, in µg. The resulting mean µg value for each sample (N = 2) is then normalized by the total protein loaded, which is used to calculate the percent of relative glycosylation (Suzuki et al. 2011)

### Data normalization and percentage calculation

As described above, levels of glycosylated αDG and core αDG are determined by interpolation against a calibration curve of control TA. To compute the percentage of core and glycosylated αDG, the mean interpolated µg value for each sample is further normalized by the total protein concentration.

$$\%\, \mathrm{control\, \alpha DG}=\frac{\mathrm{Interpolated\, value }(\mathrm{\mu g})}{\mathrm{Total\ Protein\, loaded }(\mathrm{\mu g})}*100$$

## Results

### Establishment of a suitable assay system

Therapeutic effects on dystrophin expression in muscular dystrophies like Duchenne muscular dystrophy have been directly examined in skeletal muscle in various clinical trials (Alfano et al. 2019; Charleston et al. 2018; Barthelemy et al. 2020). For conditions with distal limb symptoms, such as LGMD2I/R9, the tibialis anterior (TA) is the preferred muscle biopsy site due to its histological and functional susceptibility to the disease (Joyce et al. 2012). The TA is a practical choice for biopsy as it has identifiable physical landmarks and can be easily accessed with a fine core needle (Barthelemy et al. 2020), reducing patient discomfort. The application of the FNA method for TA extraction, which results in the limitation in tissue quantity, necessitates a customized assay methodology for the detection of αDG levels (Iachettini et al. 2015). A sensitive and reliable approach is necessary, particularly when working with patient sample biopsies of LGMD2I/R9, which exhibit severely reduced, broad range of glycosylated states of αDG.

**Fig. 1:**
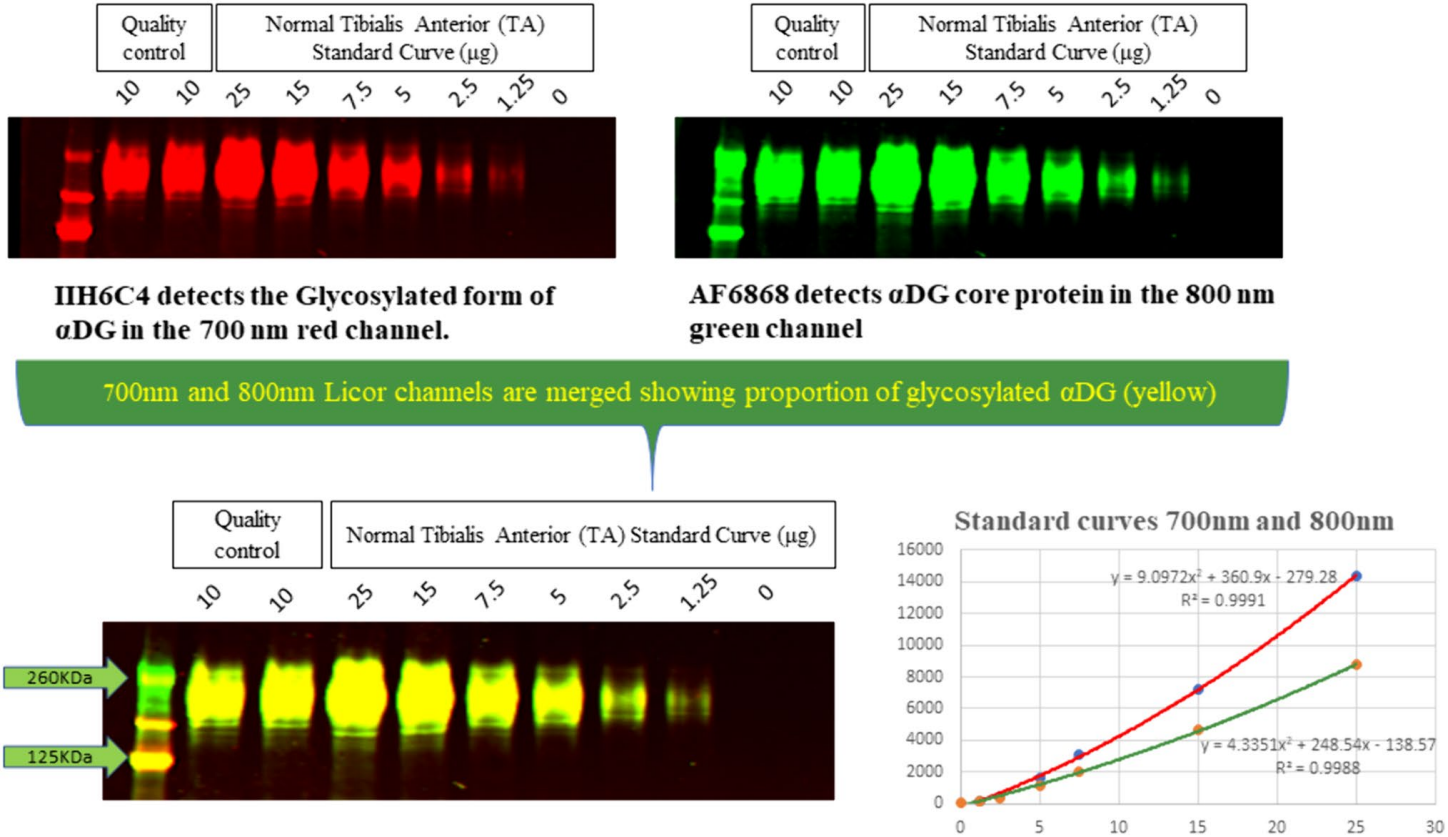
Representative Western Blot with Calibration Curve and Positive Control. Top panels show the individual channels at 700 and 800 nm in the molecular weight range of 125–260 kDa for a calibration curve and QC control generated from donor TA. The lower left panels show the merged signals for the same blot and, the bottom right plot shows the two signal intensities versus protein load.

Classical Western blotting (WB) can yield variable results due to its non-quantitative ability, only indicating the presence of the specific protein. . Other potential issues include off-target antibody binding and the limitation of detecting only specific proteins. Moreover, complications are usually seen with ECL detection, such as bands obscured in overexposed areas. Keeping this in mind, a novel, multiplexed, fluorescence-based WB method was developed that analyzes the αDG glycosylation state by simultaneously measuring core and glycosylated αDG in TA biopsy samples from patients with LGMD2I/R9. The protein extraction method from muscle biopsies uses an SDS-urea buffer for improved homogenization, which resulted in increased total protein yield and sharper protein bands. The higher efficacy of SDS-urea buffer compared to non-denaturing, nonionic detergent for extracting muscle biopsy for αDG determination was demonstrated previously (Peach et al. 2015). The selection of IIH6C4 and AF6868 antibodies was substantiated by multiple investigators (Briggs et al. 2016; Alhamidi et al. 2017; Lee et al. 2019; Cataldi et al. 2018; Wu et al. 2022; Willis et al. 2022). It was determined that AF6868 recognizes both the α and β subunits of rhDG and was therefore selected for use in the WB assay. The assay uses a more quantitative and reliable approach by a fluorescence-based detection method which has the advantage of capturing a broader dynamic range with improved reproducibility in contrast to ECL. Additionally, fluorescence-based dyes enable multiplexing by allowing simultaneous detection of multiple proteins, based on the different excitation and emission wavelength of the dyes, thus emitted light of distinct frequencies that can be individually analyzed. A calibration curve derived from control TA was used to determine the relative amounts of αDG in the patient samples and this interpolation method effectively corrects for inter-assay variability associated with antibody performance and the transfer process from gel to blot. To ensure the reliability of the results, quality control measures were implemented to monitor both inter-assay and intra-assay performance (Fig. 1).

### Evaluating antibody specificity in a multiplexed system

**Fig. 2:**
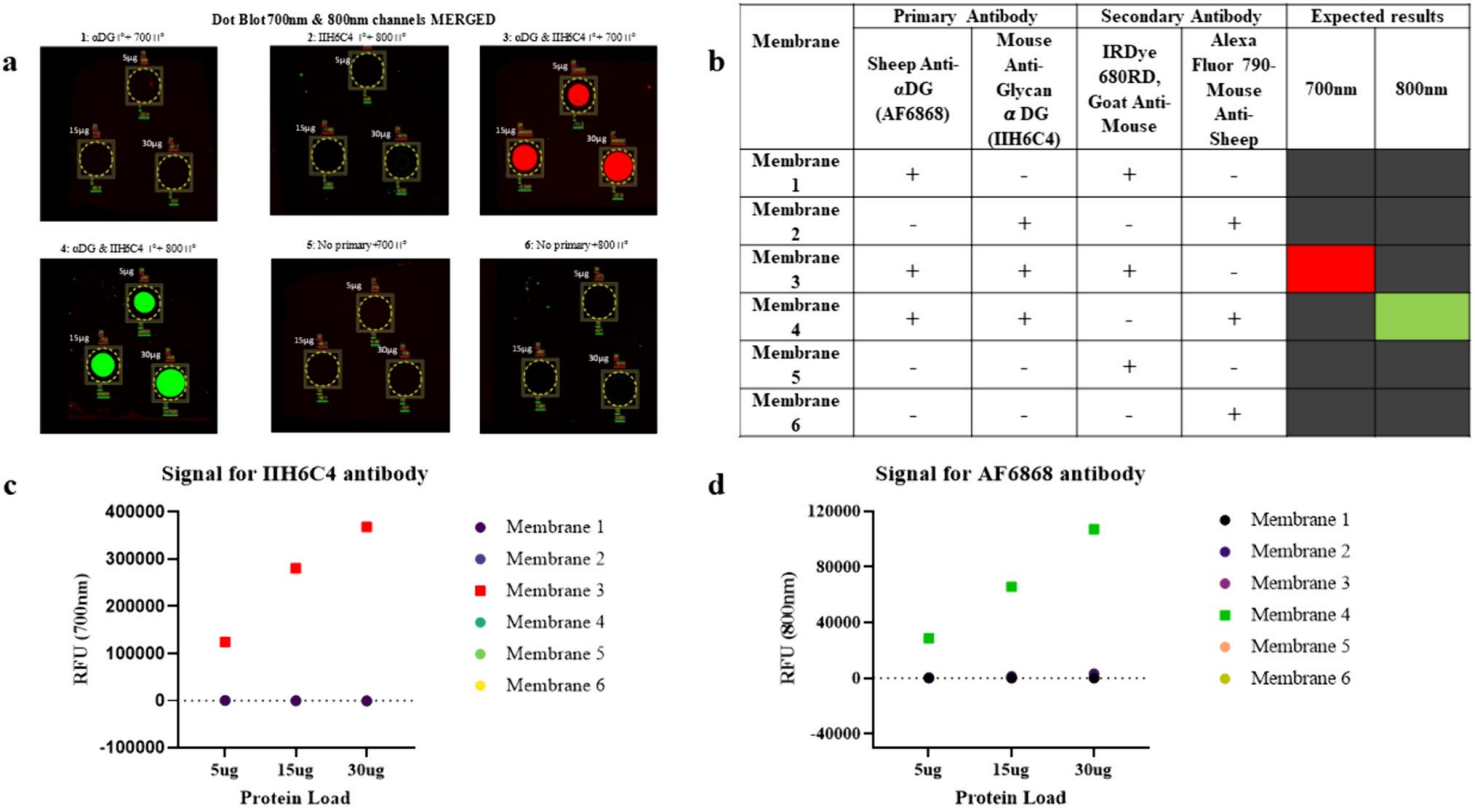
Dot Blot Analysis for Testing Cross-Reactivity. **a** 700 nm signal intensity from the IIH6C4 antibody, which binds to the unique O-mannosyl glycoepitope in the matriglycan chain of αDG; 800 nm signal intensity from the AF6868 antibody binding to core αDG; **b** Table summarizing the conditions explored to assess cross-reactivity; **c**–**d** are plots of the raw signal for IIH6C4 and AF6868 respectively. No cross-reactivity is indicated by the absence of signal at varying concentrations, whereas a positive response is indicated by concentration dependent increase in RFU.

The detection of each form of αDG requires exposure to matching pairs of primary and secondary antibodies. Because of the excess of other proteins, in particular albumin and fragments of immune globulin, in the muscle extracts, inappropriate recognition by the secondary antibodies in western blot analysis can potentially lead to increased background signal (Miyara et al. 2016). To evaluate this background (cross-reactive) reaction, varying amounts of muscle lysate, 5, 15, and 30 µg, were pipetted dropwise onto a membrane and exposed to multiple combinations of secondary and primary antibodies. The linear increase of signal intensity with the amounts of muscle lysate applied to the membrane indicates that the ratio of primary and secondary antibodies is appropriate for the amount of protein applied. Further, the specificity of the secondary antibodies to primary antibodies is demonstrated by the absence of signal when non-matching antibodies are mixed at varying lysate concentrations. The linear increase of signal intensity with the amounts of muscle lysate applied to the membrane indicates that the signals are proportional to the total protein loaded. For example, the secondary antibody for the 700 nm channel is specific for mouse IgM, which is the isotype of the IIH6C4 primary antibody. When this secondary antibody was mixed with the AF6868 primary antibody, which was a sheep IgG, no signal at 700 nm was observed because there was no binding between the incompatible primary and secondary antibodies. Similarly, the secondary antibody for the 800 nm channel is specific for sheep IgG, which is the isotype of the AF6868 primary antibody. If this secondary antibody was mixed with the primary antibody IIH6C4, which was a mouse IgM, no signal at 800 nm was reported, confirming no binding between the secondary and primary antibodies. When these antibodies are present in the right combination, they give a positive signal, proportional to the total protein loaded (Fig. 2).

### Validating antibodies using negative controls

The assay specificity was further assessed with lysates prepared from a commercially available *DAG1* knockout human embryonic kidney (HEK) 293 T cell line (lacking dystroglycan) and a wild-type HEK293T control cell line analyzed at 20 μg total protein load for the presence of core αDG and glycosylated αDG. Overall, reduced signal intensity for both channels (700 nm for glycosylated and 800 nm for core αDG) was observed in the *DAG1* knockout cell lysate compared with the wild-type cell line (Table 2).

**Table 2:** Raw signal intensities for DAG1 KO and WT HEK293T cells

Sample	N	700 nm channel RFU (IIH6C4)	800 nm channel RFU (AF6868)
Hu *DAG1* KO HEK293T (20 µg total protein)	1	385	467
Hu WT HEK293T (20 µg total protein)	1	1,390	1,960

*KO *knockout; *RFU *relative fluorescence units; *WT *wild type; *Hu* human

Separately, a negative validation control for the IIH6C4 antibody using human recombinant αDG expressed in myeloma cells (with a reduced molecular weight of 70 kDa as expected for hypoglycosylated protein), showed significantly reduced 700 nm:800 nm signal ratios, with the core αDG relatively higher (~ six fold) intensity than glycosylated αDG intensity further confirming the suitability of both IIH6C4 and AF6868 antibodies (Table 3).

**Table 3:** Raw signal intensities for recombinant αDG protein

		700 nm channel RFU (IIH6C4)				800 nm channel RFU (AF6868)			
Sample	N	Mean	SD	Median	IQR	Mean	SD	Median	IQR
Negative validation control (100 ng protein)	9*	10,483	2,426	11,200	3,680	63,300	6,465	64,100	5,900

*KO* knockout; *IQR* interquartile range; *SD* Standard Deviation; *RFU* relative fluorescence units; *WT* wild type

^*^Experiment was done by 2 analysts over a period of 3 months

### Evaluation of inter-subject variability of αDG in TA samples for calibration and quality control.

**Fig. 3:**
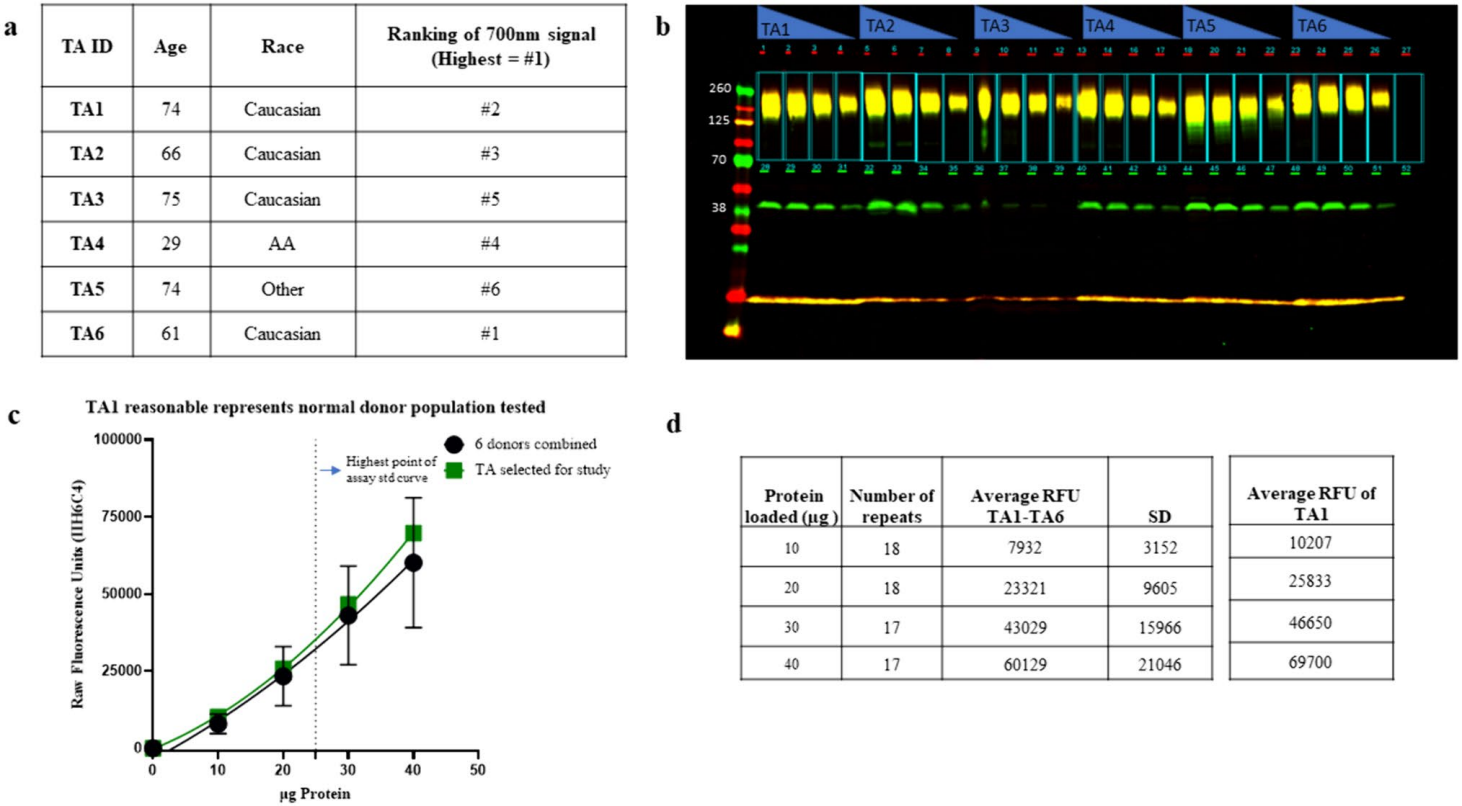
Inter-donor variability in normal human TA samples. **a** Table describing the demographics of the normal (non-affected) donors and the ID used on the WB. **b** WB image captured by Li-COR system shown with both 700 and 800 nm channels merged. **c** Plot of the 700 nm signal for glycosylated αDG at protein concentrations of 10, 20, 30, and 40 μg. TA1 is plotted separately to show the signal intensities are comparable to the average signal of all six donors. **d** table summarizing the average signal from 6 Tas at different concentrations as well as the average signal from TA1.

Analysis of TA muscle lysates from six non affected donors (Fig. 3) was conducted to explore the variability of banding patterns and the levels of αDG expression. The data demonstrated that glycosylation did not appear to be age-dependent in the six samples evaluated, which ranged from 29 to 75 years. The banding pattern was consistent among all six donors, with αDG protein bands observed at the ~ 160 kDa molecular weight range, as anticipated for fully glycosylated protein. The average signal intensity of TA1 was comparable to that of all six donor TA samples across three blots at four different concentrations. It was important to identify a balance between tissue availability and performance. The overlap in signal intensity and the sufficient availability of TA1 enabled its use as control in multiple assays across different studies, thereby minimizing inter-assay and inter-study variability.

### Assay validation

This study included validation of a WB blot assay for the simultaneous detection of glycosylated αDG containing matriglycan, the functional form of αDG, and core αDG in lysates from muscle biopsies. The small abundance of dystroglycan in muscle biopsies from patients required optimization of protein solubilization (Peach et al. 2015) for an improved protein yield and, therefore, SDS-urea buffer was chosen as it satisfied these requirements. Previous lysis condition which utilized a commercial RIPA buffer was found to be inefficient in extracting membrane bound proteins. As described previously, comparison of extraction methods for the detection of the fluorescent signals from selective antibodies were collected by a two channel LI-COR WB imager. A calibration curve for the assay was  generated using incremental dilutions of TA lysate into lysis buffer. Validation of the assay was performed using the key parameters proposed in the Bioanalytical Method Validation Guidance for Industry from the FDA (FDA guidance) which included: (1) assay precision, which is the degree of agreement among repeated measurements of the same sample under the same conditions. (2) accuracy, which is the closeness of agreement between the measured values and the true values of the analytes; (3) sensitivity, which is the ability of the assay to detect low levels of the analyte; (4) linearity which is the ability of the assay to produce results that are directly proportional to the concentration of the analyte within a given range; (5) range which is the interval between the upper and lower levels of the analyte that can be measured by the assay with acceptable accuracy and precision; and (6) robustness, which is the ability of the assay to maintain its performance under minor variations in environmental or operational conditions. Every assay gel incorporated a calibration curve, negative control (for IIH6C4 antibody) and positive controls prepared from the selected normal donor TA tissue. The fluorescence signal intensities for each tissue lysate and Quality Controls (QCs) were converted to masses using the calibration calibration curve. This allowed for the interpolation of the relative control levels of αDG and then normalization to total protein loaded for estimation of % control levels.

### Optimal model for the calibration curves

**Fig. 4:**
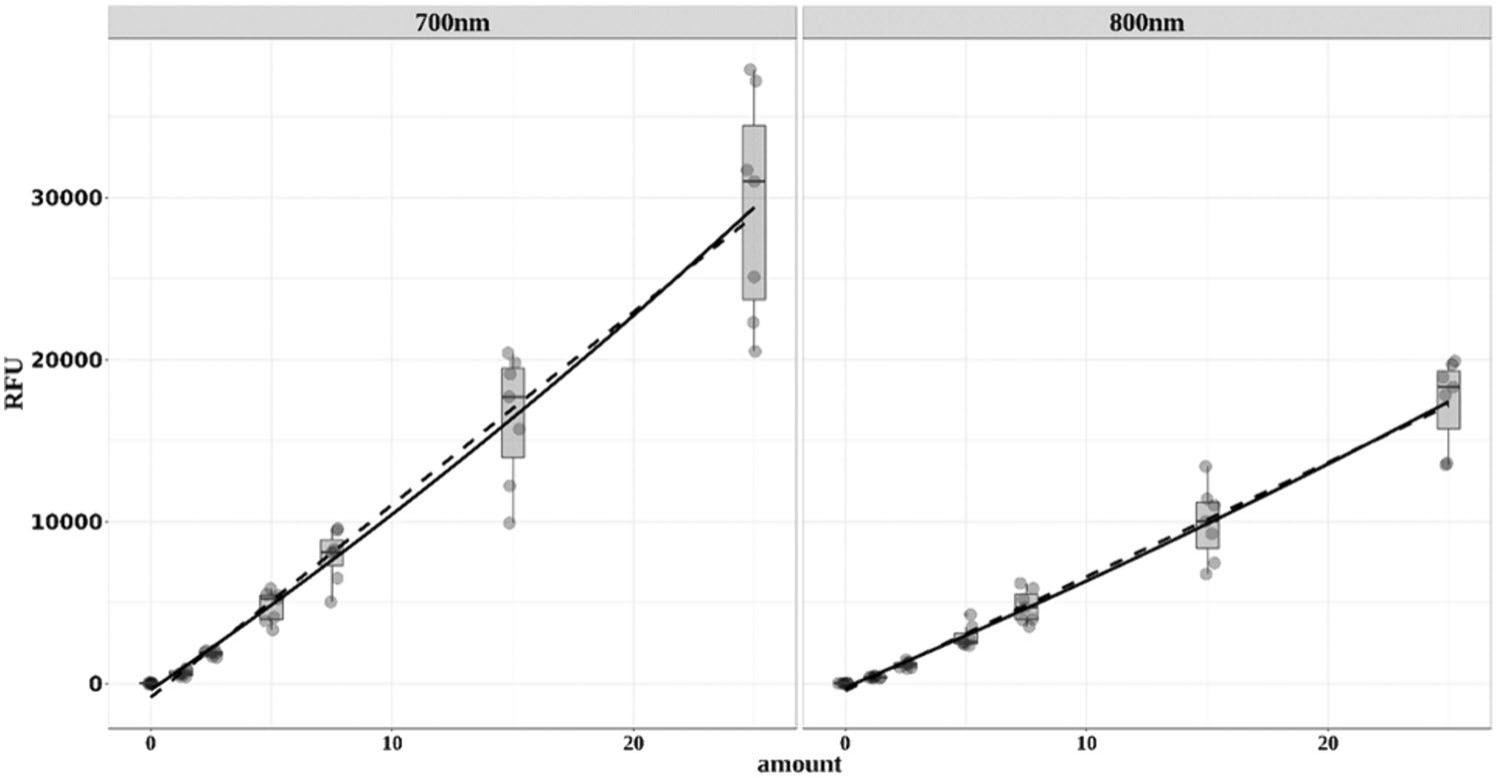
Fluorescence signals at 700 and 800 nm from the standards of seven different Western Blots. The Y axis depicts the relative fluorescence units (RFU), and the X axis depicts the amount of total protein in µg. RFU = relative fluorescence units. The fluorescence signals of calibration curves from seven WB are plotted versus the amount of total protein loaded. The solid line results from regression to a quadratic equation and the dashed line from that to a linear equation. The individual data points are summarized in boxplots with the middle line representing the median. The equations for the linear regression are RFU = -859(-451) + 1190(705) amount and for the quadratic equation RFU =  −398(−248) + 1012(627) amount (µg) + 7.2(3.2) × amount (µg) ^2^. The values in parentheses represent the regression results for the 800 nm channel.

In the absence of purified core and glycosylated αDG, a calibrant was generated from a serial dilution between 0–25 µg control TA lysate from a normal donor. The signal to concentration response was established by regression to a linear or quadratic model. Since small variations in the procedure significantly affect the electrophoretic transfer and retention of proteins on the blotting membrane, a calibration curve was required on each blot. This is evident from the spread in values in Fig. 4 and the variation in the parameters for the regression curves. The quadratic model accommodates the data better than a linear fit, consistent with the lower AIC (a measure for information not explained by the model) value and in most cases with the calculated p < 0.05 for the quadratic term (Table 4). Furthermore, since the p values for the intercepts obtained from regression are not significant in most cases and the confidence intervals cross zero, it could be set to the physically sensible value zero. However, “nonproportional” concentration response curves with intercepts different from zero have been reported by other authors (Butler et al. 2019; Pitre et al. 2007) for WB. Interestingly, previous reports suggest WB calibration curves fit to polynomial or exponential terms (Pitre et al. 2007). For quantitative evaluation of the blots, the relative amounts in µg of core and glycosylated αDG, in terms of total protein in the samples, are determined by interpolation using the inverted calibration curve of the signals measured with the protein standard mixture (Heidebrecht et al. 2009; Suzuki et al. 2011).

### Background signal and limit of detection (LOD)

**Fig. 5:**
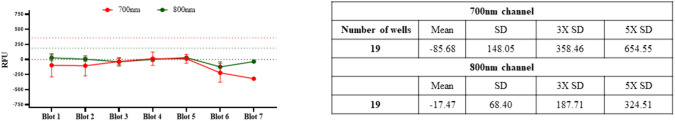
Signal Trend in Blank Wells. Left-hand side is a Levi-Jenning’s plot showing the signal intensities (solid lines) for blanks across 7 blots. Each blot is N = 3 except for Blot 7 (N=1). The red dotted line is 3X Standard Deviation of the 700 nm background signal. The green dotted line is the 3X Standard Deviation of the 800 nm channel; The table on the right summarizes data from 7 blots.

The stability of the blank signal in the assay is illustrated in a Levey-Jennings plot (Fig. 4) of the measured signals versus the gel number. The samples consist of protein-free buffer which are analyzed in an immunoblot along with the samples used for calibration of the assay. The mean and standard deviation of all background wells across all empty lanes were calculated and are shown in Fig. 4. The LOD was derived using the noise level above the blank by determining the signal of 3 times the standard deviation. The results of the estimation and the inverse predicted values are summarized in Fig. 5.

### Evaluating assay sensitivity and lower limit of quantitation

**Fig. 6:**
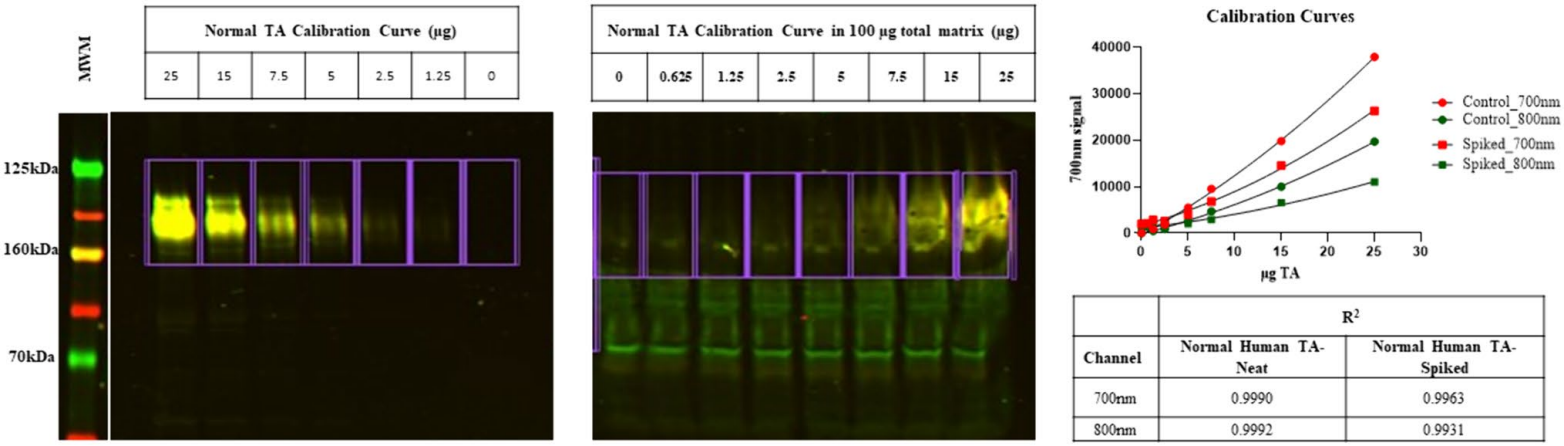
Assay Sensitivity in a fixed 100µg Tissue Matrix. MWM = molecular weight marker. Blot has been split to show only relevant data. 700 nm and 800 nm channels are merged for the same blot. On the right is a plot of the 700 nm signal intensities, which correspond to matriglycan and the 800 nm signals corresponding to core αDG, versus the total amount of spiked in protein. The solid lines correspond to a fit with a quadratic equation. The inset table describes the coefficient of determination (R^2^) for both the spiked in and neat calibration standards.

An initial assessment of LGMD2I/R9 patient samples indicated that a high amount of protein is necessary to obtain a quantifiable signal of αDG. This necessitated an evaluation of the assay’s sensitivity at a matrix concentration of 100 µg. In the absence of a suitable matrix deficient in glycosylated αDG, TA lysate from a transgenic mouse with an *FKRP*-P448L mutation (gift from Dr. Qi Long Lu, Atrium Health), which results in severely hypoglycosylated αDG, which runs at a molecular weight lower than that of normal human control, was used to assess assay sensitivity. In a matrix containing 100 µg total protein from both mouse and human TA, varying amounts of normal control TA lysate was added to evaluate the sensitivity range. As shown in Fig. 6, a consistent increase in signal was observed across the high matrix TA calibration curve. The difference in slope is attributed to possible interference from the inherent glycosylated αDG signal from the mouse matrix tissue. Further, the consistency of glycosylated αDG /core αDG signal ratio was also reflected with a coefficient of variation (CV) of less than 30% and an overall *R*^2^ of  > 0.99 from a quadratic fit. In the previously described HEK293T cells , the signal for glycosylated αDG at 700 nm was found to be lower than that for the core protein at 800 nm for the wildtype. This contrasts with the trend in the calibration curves, where the 700 nm signal is higher than the signal at 800 nm at the same protein load. It is worth noting that HEK293T cells very inefficiently produce the critical mucin domain, which is required for matriglycan formation. Therefore, signal from this cell system can be assumed to differentiate between the presence and absence of the critical epitope (Sun et al. 2022; Harrison et al. 2012). Further analysis of the calibration located the detection limit at roughly 1 µg.

### Evaluating inter and intra assay precision

**Fig. 7:**
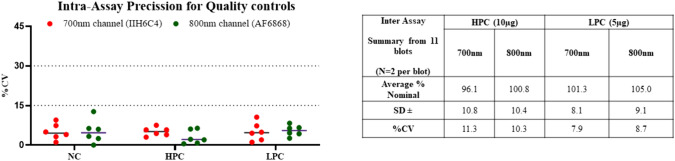
Results from Inter- and Intra-assay precision. Left: intra assay variability based in absolute signal for Negative Validation Control (NC), High Positive Control (HPC) and Low Positive Control (LPC); Right table is the inter assay variability based on interpolated percent nominal values of quality controls for HPC which was tested at 10 µg total protein and LPC which was tested at 5 µg total protein

Inter- and intra-assay precision was evaluated by collecting the raw fluorescent signal intensities for test and control samples. To examine intra-assay operator precision, two analysts ran each of the validation controls in duplicate and on six gels on different days. For assay precision determination, each of two analysts ran TA lysates at 10 and 5 µg per lane in quadruplicate on a gel for a total of six gels across multiple days. Assay performance is monitored using quality controls from the same donor. Intra-assay precision is evaluated using a negative control of purified protein and positive controls of TA lysate. Signal variability between sample replicates is assessed. For inter-assay precision, interpolated (back-calculated) values of the positive controls were tracked. Results demonstrate good assay reproducibility for both 700 and 800 nm channels with a coefficient of variation below 15% (Fig. 7).

### Patient sample analysis, banding patterns and gating differences

**Fig. 8:**
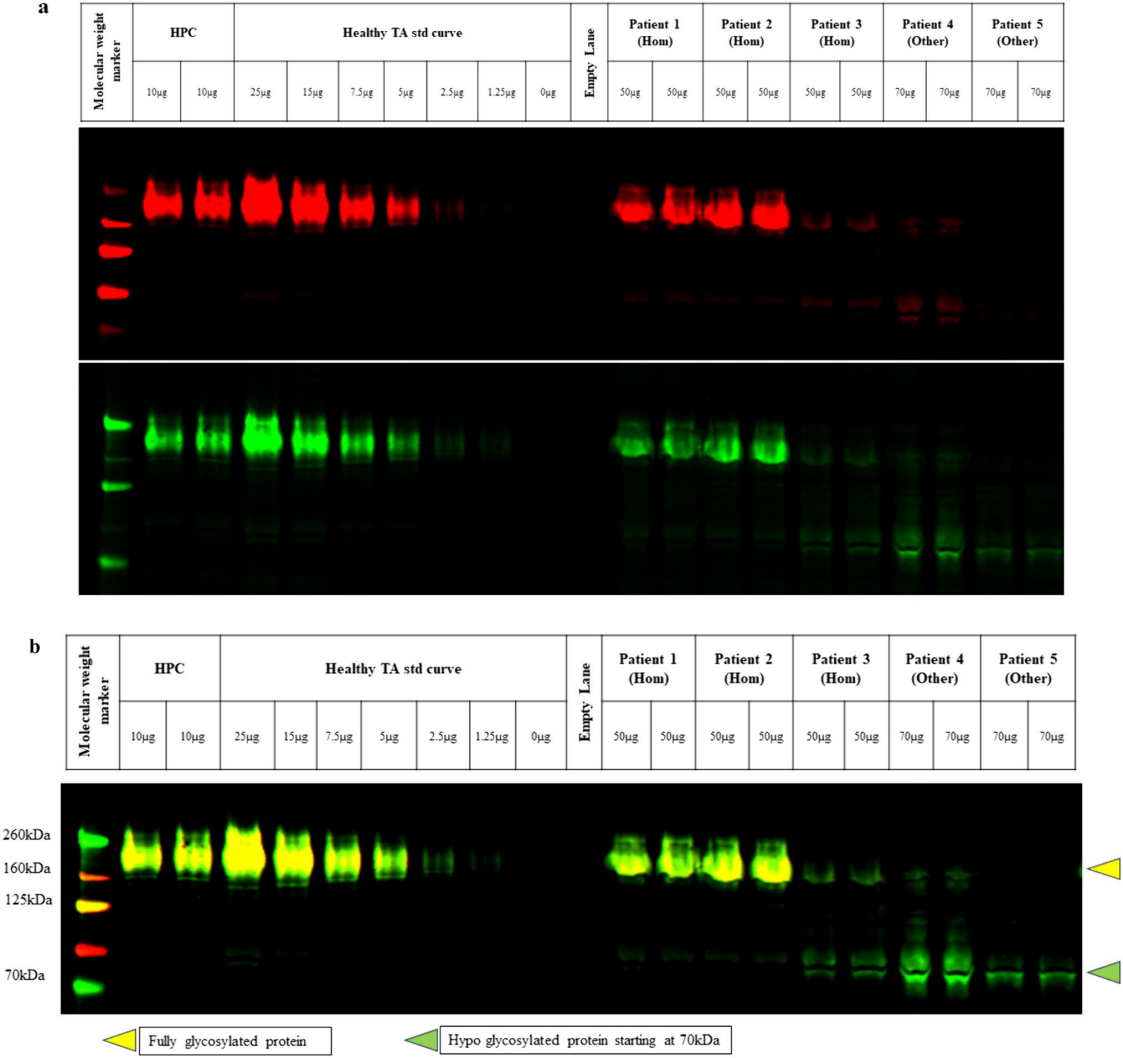
Blot from a Natural History Study in Patients with the Common Homozygous L276I Mutation and Other *FKRP* Genotypes. “Hom” indicates patients homozygous for L276I mutation; “Other” indicates patients with compound heterozygous mutations with either L276I/Other or Other/Other FKRP mutation. Panel **a** Is the same blot when imaged under separate 700 and 800 nm channels. Panel **b** is the Blot with merged channels. As described previously, a perfect overlap of channels results in yellow bands. Fully glycosylated protein is expected to migrate at 160 kDa molecular weight. Decreases in glycosylated αDG (faint yellow band) at the 160 kDa molecular weight region and reduced molecular weight of αDG core for patient 2 with other *FKRP* mutation, which appears as multiple bands spanning the ROI of 70 to 160 kDa, suggests hypoglycosylation of αDG.

**Fig 9:**
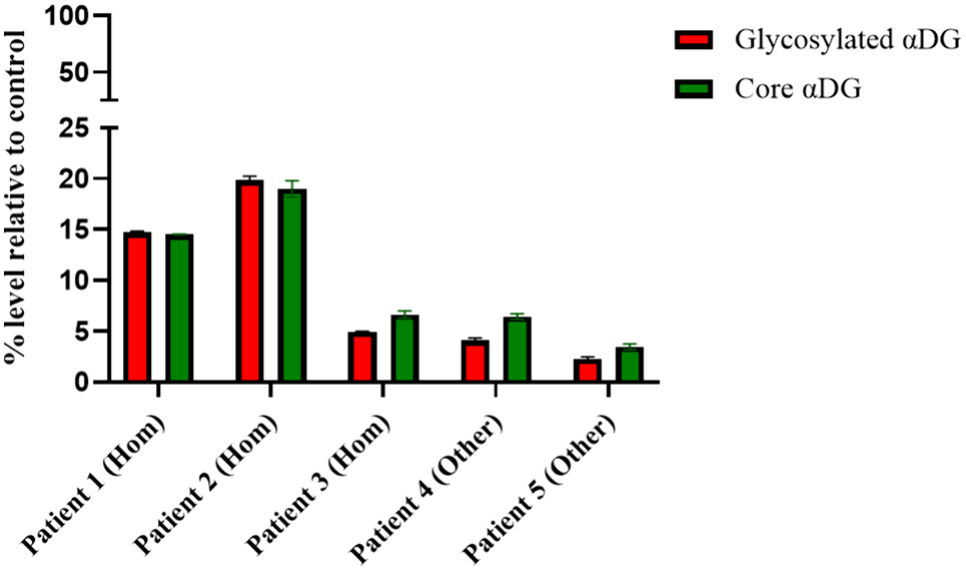
Relative levels of αDG in TA biopsies of patients with LGMD2I/R9. The figure presents relative levels of αDG from patient biopsies in Fig. 8a, b. The relative percent levels of αDG were determined from the 700 nm signal (for glycosylated αDG) and 800 nm signal (for core αDG) were interpolated to the calibration curve. The resulting relative µg amount was normalized to the total protein loaded.

The validated assay was used to evaluate levels of glycosylated and core αDG in patient biopsy samples. A calibration curve was generated with normal human TA samples to allow simultaneous quantification of glycosylated and core αDG levels, and samples were run in duplicate on each blot, including the positive control to assess assay performance. The Region of Interest (ROI) is a standardized area on the blot, specifically chosen for the detection and analysis of the protein under study. This region is selected based on the target protein’s molecular weight and the separation achieved during gel electrophoresis. The ROI plays an important role in the precise quantification and analysis of WB. It determines data accuracy and reproducibility and facilitates the comparison of protein expression across samples tested in varying quantities (Taylor et al. 2013; Bass et al. 2017; Aldridge et al. 2008). The blot shown in Fig. 8 includes three patients with homozygous L276I/L276I genotypes (Patients 1–3) and two patients with other *FKRP* genotypes, participating in the natural history study (MLB-01–001; NCT04202627). The banding patterns observed for the L276I/L276I patients 1 and 2 resemble the normal control, but that of patient 3 exhibits a greater heterogeneity of glycosylation patterns. It is interesting to note that L276I/L276I patient 3 exhibits a broader range of hypoglycosylated αDG bands, with molecular weights between 70–160 kDa, compared to patients 4 and 5 who have other *FKRP* genotypes, suggesting the presence of a broad spectrum of glycosylation pattern in LGMD2I/R9 patients compared to normal donors (Fig. 9).

## Discussion

This report presents the development and application of a novel multiplexed fluorescence-based WB method for the quantitative determination of glycosylated and core αDG in skeletal muscle biopsies. For validation samples from normal donors and patients with LGMD2I/R9, a disease characterized by chronic hypoglycosylation of αDG, were compared. LGMD2I/R9 patients have mutations in the fukutin-related protein (*FKRP*). Partial loss of function in the FKRP enzyme leads to incompletely glycosylated (“hypoglycosylated”) αDG, which then fails in its critical role of stabilizing muscle contraction and maintaining muscle cell membrane integrity (Georganopoulou et al. 2021; Endo 2015). The developed assay can distinguish between reduced glycosylation levels of αDG from compound heterozygous LGMD2I/R9 patients expressing both L276I and another defective variant of FKRP. Since the readout of the assay is directly linked to the molecular defect, it may be considered a marker for the severity of disease and potentially be used to interrogate effects of therapeutic intervention (Godfrey et al. 2007). However, it should be remembered that correlation of the degree of glycosylation of αDG as a biomarker for disease severity and for monitoring disease modifying treatment is not yet well established in the literature (Johnson et al. 2018).

Several studies have used IHC for the detection of glycosylated and core αDG in samples of muscle tissues biopsies (Fritschy 2008; Johnson et al. 2018); comparative evaluation of protein expression is difficult due to variability of protein staining, lack of reusable calibration controls and limited detection range (Fritschy 2008). Similarly, the analysis of muscle lysate by MS techniques is not suitable for quantitation of core and glycosylated protein as they require complex pre-purification steps and analysis of glycosylation states of αDG protein is difficult to perform (Chandel and Campbell 2023; Harrison et al. 2012). Enzyme-linked immunosorbent assays (ELISA) are another tool for the quantitation of biomarkers, but available commercial assays do not differentiate between various glycosylated forms of αDG, and tissue lysis buffer interferes with ELISA detection (Crowe et al. 2016). Thus, Western blotting appears to be most appropriate, as it can be adapted for quantitative analysis of complex protein mixtures and the ability to track specific molecular weight(s) provide potentially significant information for diagnosis of hypoglycosylation. In addition, combined with fluorescence detection WBs permit multiplex detection (Anderson and Davidson 1999; Schutz-Geschwender et al. 2004).

The primary antibodies, IIH6C4 and AF6868, and their utility in muscle biopsy assessment have been widely discussed for the detection of glycosylated and total αDG, respectively (Michele et al. 2002; Muntoni et al. 2011). The monoclonal antibody IIH6C4 has been used to monitor the increase of matriglycan content in αDG in FKRP deficient mice upon treatment with ribitol over time (Cataldi et al. 2018) and its reduction due to glycosidase treatment in vitro (Chandel and Campbell 2023). An alternate antibody, VIA4-1, has been used for the detection of glycosylated αDG in WB of tissue extracts from LGMD patients. In contrast to VIA4-1, IIH6C4 can prevent the binding of glycosylated αDG to laminin and may be considered a functional surrogate of the native ligand binding. Accordingly, as the impaired ability of αDG to interact with laminin is thought to be the cause of LGMD, the signal resulting from IIH6C4 binding may have a correlation to disease severity. Thus, the pairing of IIH6C4 and AF6868 appears to be the appropriate choice to monitor the levels of glycosylated (and therefore functional) versus total αDG in patients.

As the assay is developed for the analysis of fine needle biopsies, the small sample size required effective and optimal tissue lysis prior to separation on gels. Efficient recovery of the proteins is particularly important for patient samples due to the low abundance of total and specifically glycosylated αDG. In the present study, samples from tibialis anterior were processed for evaluation. TA is one of the most active lower leg muscles and is considered optimal for biomarker studies in other muscular diseases (Carberry et al. 2012; Joyce et al. 2012; Iachettini et al. 2015). From a practical perspective of obtaining tissue biopsy samples from patients, TA has consistent physical landmarks and is relatively easy to biopsy using a small core needle, thereby limiting patient burden. During WB development, comparison of different buffer systems for extraction of proteins from TA confirmed the higher efficiency of denaturing mixtures composed of SDS/urea and a reducing agent (Zardini et al. 1993) relative to commercial buffers containing nonionic detergents. In the context of the present study with dystroglycan, the ability of the buffer system to dissociate and solubilize large protein complexes with laminin and myosin is particularly advantageous (Anderson and Davison 1999; Miskiewicz and MacPhee 2019). Low abundance of dystroglycan protein requires large volumes of sample to be separated by the gel. Under these assay conditions, in contrast to buffers with SDS, a concomitant excess of nonionic detergents disrupts the protein migration.

Most commonly proteins are detected on WB either by ECL or by fluorescence intensity. In contrast to fluorescence signals, ECL detection is sensitive to small variations in the execution of the assay, has a narrow dynamic range and often does not produce a linear response proportional to the target antigen. ECL chemistry at the basis of the reaction depends on timing and substrate availability (Pillai-Kastoori et al. 2020). Early reports on the abundance of αDG between unaffected population and patient samples varied widely, the extended dynamic range of fluorescence detection compared to that of ECL makes it particularly attractive for comparative studies related to LGMD. Moreover, because of the reduced variability relative to ECL, fluorescence detection appears to be more suitable for serial experiments on patient samples. The reported WB assay is based on the detection of glycosylated relative to total αDG on the same blot. This allows for the possibility of simultaneous detection rather than a need for error-prone stripping of blotting membranes required for ECL. The use of fluorescence detection to quantitate a muscle protein of clinical interest has also been reported for dystrophin, a protein implicated in a disease similar to LGMD (Schnell et al. 2019).

With only 0.0013% (89 nmol/1 g mouse skeletal muscle extract) αDG expected in tissue extracts (Johnson et al. 2013), matrix effects and cross reactivity may pose a potential problem. The selectivity of both secondary antibodies was demonstrated by the absence of signals, when mismatched pairs of primary and secondary antibodies were tested in a multiplexed dot-blot assay. The selectivity of the primary antibodies for their specific epitopes was inferred by a fivefold difference in signal between HEK cell extracts from dystroglycan knock-out and wild type mice.

A protein standard for glycosylated αDG is not available as the expressed protein with complete mucin modification reactive with IIH6C4 could not be obtained from a commercial source. Thus, extracts of healthy control muscle was used to generate a calibration curve and the amount of glycosylated protein was expressed in terms of microgram of the total healthy extract. Similar quantitation of a mixture of components as relative amounts using “mixed calibration curves” have been used in HPLC analysis of plasma samples and metabolomics (Chen et al. 2017; Liang et al. 2010). As all experiments presented in the report use the same healthy lysate, the reported amounts albeit relative, are consistent among each other. Since the calibration curve is generated on each gel, fluctuations in signal intensity of αDG are normalized between individual WB and quantitative comparison is possible between gels. Comparison with the calibration curve also assures that both forms of αDG are applied to the gel in a range with signals increasing monotonically with dose, as generally recommended for quantitative WB. Although calibration curves for WB are usually presumed to be linear, statistical analysis of the presented data yielded a better fit for a parabolic curve. This is consistent with some reports in literature (Butler et al. 2019). The LLOQ extrapolated from this curve equals 1 µg total protein, which translates into an estimated detection limit of 13 pg of dystroglycan in the muscle extract. Although very small, this estimate appears consistent with detection limits for fluorescent WB reported in the literature. The low detection limit compares favorably with an ECL based WB assay reported for dystrophin, which was assessed around 3 ng. Similar to ECL based assay for dystrophin, the αDG assay presented here has a precision expressed as a coefficient of variation of 30%. With its high sensitivity this WB dystroglycan assay is particularly valuable for analysis of patient samples, as they are characterized by low expression/low levels of glycosylated and total dystroglycan and a generally lower content of intact muscle fibers.

Our results of testing patient samples from compound heterozygous LGMD2I patients expressing both L276I and another defective FKRP variant indicated reduced levels of glycosylated αDG relative to their homozygous counterparts. Several reports suggest a correlation between severity of disease and level of αDG glycosylation (Mercuri et al. 2003; Brown et al. 2004) but this has been disputed by others (Jimenez-Mallebrera et al. 2009; Alhamidi et al. 2017). The discrepancies may have resulted from limitations in the quantitative interpretation due to the ubiquitous use of ECL detection and the lack of calibration, normalization procedures and differences in the muscle biopsy location. It’s unclear if the necessary correction of differences in the ECL reaction between blots could be carried out under conditions of the reported methods. In contrast, the presented approach uses a more stable detection system and enables reliable comparison, as all results are normalized to a calibration curve on each blot. Thus, consistent adaptation of the methodology of this report for evaluating glycosylated αDG and data normalization may pave the way to further understanding of hypoglycosylation and its relation to disease severity.

### Supplementary Information

Below is the link to the electronic supplementary material.

Supplementary file1 (PDF 73 KB)

## Data Availability

The data supporting the findings of this study are available within the article and/or its supplementary material.
