# Correction to: Validation of a novel western blot assay to monitor patterns and levels of alpha dystroglycan in skeletal muscle of patients with limb girdle muscular dystrophies

**DOI:** 10.1007/s10974-024-09678-4

**Published:** 2024-06-24

**Authors:** Thulashitha Rajasingham, Hector M. Rodriguez, Andreas Betz, Douglas M. Sproule, Uma Sinha

**Affiliations:** 1 Department of Preclinical/Clinical Pharmacology, ML Bio Solutions, a BridgeBio company, Palo Alto, USA; 2 Department of Clinical Development, ML Bio Solutions, a BridgeBio company, Palo Alto, USA

**Correction to: Journal of Muscle Research and cell motility**

10.1007/s10974-024-09670-y

The copyright holder for this article was incorrectly given as

This is a U.S. Government work and not under copyright protection in the US; foreign copyright protection may apply 2024

It should be replaced by

© ML Bio Solutions Inc., a BridgeBio Pharma, Inc. Company 2024

The original article has been corrected.

