# Supplementary material for: Validation of a novel western blot assay to monitor patterns and levels of alpha dystroglycan in skeletal muscle of patients with limb girdle muscular dystrophies
Source: J Muscle Res Cell Motil. 2024 Apr 18;45(3):123–38. doi: 10.1007/s10974-024-09670-y (PMC11316722; doi:10.1007/s10974-024-09670-y)
Supplement: Supplementary file 1 — Supplementary file1 (PDF 73 KB) [file 10974_2024_9670_MOESM1_ESM.pdf]

	700 nm							800 nm						
	**Run_2**	**Run_4a**	**Run_4b**	**Run_5**	**Run_6**	**Run_10**	**Run_11**	**Run_2**	**Run_4a**	**Run_4b**	**Run_5**	**Run_6**	**Run_10**	**Run_11**
**Slope (RFU/ug) (CI)**	1,298** (1,211, 1,385)	806** (728, 885)	888** (847, 930)	1,295** (1,219, 1,371)	1,490** (1,362, 1,619)	1,523** (1,415,1,632)	1,026** (968, 1,085)	836** (773, 899)	534** (488, 580)	536** (513, 560)	778** (736, 820)	715** (656, 773)	790** (723, 857)	746** (706, 787)
**Intercept (CI)**	-627 (-1,632, 378)	-663 (-1,574, 247)	-353 (-834,128)	-1,260* (-2,142,378)	-1,581 (-3,067,-95)	-1,435 (-2,693, -178)	-93 (-771, 584)	-332 (-1,062, 397)	-337 (-872, 198)	-161 (-431, 110)	-686* (-1,169, -203)	-744 (-1,419, -70)	-842 (-1,617, -67)	-55 (-525, 415)
**AIC**	120	119	110	118	125	123	114	115	111	102	110	114	116	109
**Adjusted R^2**	0.99	0.99	1	0.99	0.99	0.99	0.99	0.99	0.99	1	1	0.99	0.99	1
Notes:	*Significant at the 5 percent level<br>**Significant at the 1 percent level													

	700 nm							800 nm						
	**Run_2**	**Run_4a**	**Run_4b**	**Run_5**	**Run_6**	**Run_10**	**Run_11**	**Run_2**	**Run_4a**	**Run_4b**	**Run_5**	**Run_6**	**Run_10**	**Run_11**
**Linear (CI)**	1,476** (1,159, 1,793)	516** (367, 666)	769** (643, 894)	1,059** (847, 1,270)	966** (879, 1,053)	1,108** (934, 1,282)	1,193** (1,015, 1,371)	989** (775, 1,203)	372** (269, 474)	471** (399, 544)	696** (541, 850)	477** (437, 517)	525** (443, 607)	858** (731, 986)
**Quadratic (CI)**	-7 (-20, 5)	12* (6, 18)	5 (-0, 10)	10 (1, 18)	21** (18, 25)	17** (10, 24)	-7 (-14, 0)	-6 (-15, 2)	7* (3, 11)	3 (-0, 5)	3 (-3, 9)	10** (8, 11)	11** (8, 14)	-5 (-10, 0)
**Intercept (CI)**	-1,090 (-2,348, 169)	91 (-503, 684)	-42 (-539, 455)	-645 (-1,485, 195)	-219 (-564, 126)	-356 (-1,046, 335)	-527 (-1,234, 180)	-732 (-1,581, 117)	86 (-320, 491)	9 (-280, 297)	-473 (-1,086, 140)	-127 (-288, 33)	-153 (-478, 172)	-346 (-851, 160)
**AIC**	120	109	107	114	102	112	112	114	104	99	110	91	101	107
**Adjusted R^2**	0.99	1.00	1.00	1.00	1.00	1.00	1.00	0.99	1.00	1.00	1.00	1.00	1.00	1.00
Notes:	*Significant at the 5 percent level<br>**Significant at the 1 percent level													
